# Supplementary material for: Are carbon emissions trading and green financial instruments synergistic? -Comprehensive quantitative research based on content analysis
Source: PLoS One. 2024 Mar 7;19(3):e0298601. doi: 10.1371/journal.pone.0298601 (PMC10919601; doi:10.1371/journal.pone.0298601)
Supplement: S1 Data — (ZIP) [file pone.0298601.s001.zip › Data/地级市Z_F工作报告.zip!/║╙─╧/┬σ╤⌠╩╨/└┤╫╘╣1⁄2╓┌║┼í╛┬φ┐╦╩2╛▌═°í┐_2016.docx]

各位代表：

现在，我代表市人民政府，向大会报告工作，请予审议，并请各位政协委员和其他列席人士提出意见。

一、“十二五”及2015年主要成就

刚刚过去的五年，是洛阳发展进程中具有重要历史意义的五年。面对新世纪以来前所未有的困难形势，市政府在省委、省政府和市委的坚强领导下，在市人大、市政协的监督支持下，全面落实党的十八大和十八届三中、四中、五中全会精神，深入贯彻习近平总书记系列重要讲话精神，主动适应经济发展新常态，统筹推进改革发展稳定工作，较好地完成了“十二五”各项目标任务。

——综合经济实力持续增强。2015年，全市经济总量突破3500亿元，是2010年的1.5倍；五年平均增速高于全国1.8个百分点；人均生产总值52287元，是2010年的1.5倍。规模以上工业增加值1386.2亿元，是2010年的1.4倍。固定资产投资、公共财政预算收入、社会消费品零售总额、金融机构存贷款余额等主要经济指标均实现翻番。

——产业结构调整成效显著。五年实施亿元以上产业项目1765个，完成投资3826亿元。工业加速集聚升级。2015年，全市装备制造业主营业务收入达到2282.2亿元，硅光伏产能居全省首位，铝精深加工能力达178万吨；电子信息、机器人及智能装备制造等战略新兴产业主营业务收入占全市工业的四分之一；主营业务收入超百亿元的产业集聚区达15个、超500亿元1个。现代服务业提质增速。2015年，服务业增加值占生产总值的比重达43.7%，比2010年提高12个百分点。新增5A级景区3家。一批大遗址保护展示工程建成开放，丝绸之路和大运河申遗成功。牡丹文化节升格为国家级节会。我市入选国家电子商务示范城市。特色高效农业不断壮大。2015年，六大优势产业总面积达到450万亩，牡丹面积是2010年的8.5倍，我市荣膺“中国牡丹花都”称号。市级龙头企业、农民合作社数量分别是2010年的3倍和3.7倍，农业产业化产值占第一产业的60%。

——创新创业活力竞相迸发。五年累计投入科研经费249.5亿元，是“十一五”的2.2倍；新增研发机构685家，是“十一五”的3.4倍；专利申请量3.65万件，是“十一五”的3.4倍。国家重点实验室达到7个、占全省的50%；获得国家科技奖励26项，占全省的24.5%。高铁轴承、载人潜水器关键设备等一批核心技术实现突破。与中科院等合作成立了一批新型研发机构。培育引进“两院”院士30名、中原学者7名。洛阳大学科技园升格为国家级大学科技园。我市荣膺首批国家知识产权示范城市，连续荣获“全国科技进步先进市”称号。各类众创空间蓬勃兴起。创业担保贷款发放量连续三年排全国地级市第一。

——城乡基础设施更加完善。第四期城市总体规划获批实施。城镇化率由44.33%提高到52.65%。示范区建设稳步推进。城乡一体化示范区地区生产总值年均增长12.4%，入选国家首批智慧城市试点，建成区面积达69平方公里。核心区建成了会展中心、河科大一附院新区医院等一批标志性工程；伊滨新区主体框架拉开，骨干路网基本形成，水、电、热、气等城市功能基本具备。承载功能明显提升。中心城区新增通车里程87.6公里；新建改建干线公路599公里、农村公路2532公里；高速公路通车里程达501公里，实现了县县通高速，环市区半小时经济圈基本形成。开通国内航线6条，旅客吞吐量是2010年的2.6倍。新增110千伏及以上变电站27座，110千伏变电站实现了县域和产业集聚区全覆盖。洛阳迈入“全光网”时代。一批县城新区初具规模，重点镇、旅游名镇建设全面加速。新农村建设成效显著。为197万农村群众解决了饮水安全问题，硬化通组通户道路10337公里，66个市级美丽乡村试点顺利实施，我市入选全省改善农村人居环境示范市。生态环境更加宜居。完成大气污染治理重点工程929项，燃煤机组超低排放全省领先，水环境质量100%达标。伊滨公园等生态工程建成投用，伊洛河水生态文明示范区等项目进展顺利。创建省级以上生态县、乡镇（村）329个，栾川成为全省首个国家级生态县。我市荣膺国家森林城市、全国水土保持生态文明城市称号。

——改革开放步伐不断加快。成功争取了新型城镇化、信息惠民、养老服务业综合改革、PPP模式试点等一大批国家级试点（示范）机遇，入选财政部第二批PPP示范项目11个、居全省第一。商事制度改革成效显著。行政审批事项压缩59%，非行政许可审批全部取消。事业单位配套改革及法人治理结构试点工作、38家重点企业改革改制任务全面完成。公立医院改革取得重大突破。新增上市挂牌企业29家。民营经济占生产总值的比重提高10个百分点。引进境内外500强企业21家。实际利用省外境内资金2533.4亿元、外资109.5亿美元，分别是“十一五”的3.4倍和2.8倍。洛阳成为丝绸之路经济带主要节点城市，一拖集团首批入驻中白工业园，中铁隧道集团在乌兹别克斯坦承建了中亚第一长隧。

——人民群众得到更多实惠。2015年，全市各级财政民生支出占财政总支出的73%。城乡居民人均可支配收入分别达到28686元和10667元，是2010年的1.6倍和1.9倍。五年新增城镇就业61.3万人、再就业21.8万人，新转移农业劳动力23.9万人次，城镇登记失业率控制在4.5%以内。在全省率先实行社会保险“五险合一”。洛阳妇幼保健院完成搬迁，市区医院新增床位6866张，城乡15分钟医疗服务圈基本形成。提前实现城乡居民养老保险制度全覆盖。建成保障性住房8.8万套。农村贫困家庭危房改造数全省第一。31万人稳定脱贫。新建改扩建中小学、幼儿园931所，幼儿园总数增长1.2倍。洛阳师范学院搬迁全部完成，3所职业院校成功升格，普通高招上线人数连创新高。图书馆、文化馆全部免费开放，公共文化服务网络、城乡健身设施实现全覆盖，广播电视覆盖率达到97%。人大代表建议、政协提案办理工作取得新成效。平安洛阳建设扎实推进，安全生产形势总体平稳，社会大局保持稳定。

2015年是“十二五”的收官之年。全市上下认真贯彻党中央国务院和省委省政府一系列决策部署，坚持调中求进、改中激活、转中促好、变中取胜，圆满完成了各项目标任务。地区生产总值同比增长9.2%，居全省第4位，增速重回全省第一方阵；粮食总产50.74亿斤，夏秋粮单产、总产均创历史新高；固定资产投资增长18.6%，规模以上工业增加值增长9.7%，一般公共预算收入增长10.2%，社会消费品零售总额增长12.3%。重点抓了以下工作：

（一）全力以赴稳增长、保态势，经济运行稳中向好。加大企业服务力度。千方百计帮助企业协调解决实际问题，中集凌宇、一拖集团等企业产品列入政府采购名录，中航锂电、中航光电、725所等一批创新型企业产值大幅增长。银企洽谈会签约资金724.6亿元，落实率达到96.8%。企业还贷周转金全年周转额达到52.1亿元，节省企业融资成本6000多万元。持续增加有效投资。麦达斯轨道铝型材一期、中信重工高端电液智能控制装备制造一期等一批项目竣工投产，双瑞特种装备产业基地、惠普软件人才产业基地、中兴通讯智慧产业基地等一批项目加快建设。千方百计扩大外贸。获批省级出口基地2家。完成进出口总额121.5亿元,增长12%。与“一带一路”沿线国家实现贸易总额8.34亿美元，增长31.1%。实际利用外资25.5亿美元、省外境内资金661亿元。

（二）坚持不懈调结构、促转型，产业升级亮点纷呈。工业结构持续优化。实施重大工业结构调整项目405个，完成投资612亿元。洛阳石化1800万吨炼油扩能改造前期工作大头落地。中信重工重装高科产业基地、利尔中晶光伏产业园加快建设。我市成为全省唯一的机器人及智能装备产业基地。六大高成长性产业增加值同比增长13.9%，高于全市工业4.3个百分点。现代服务业不断壮大。接待游客突破1亿人次，旅游总收入779.9亿元，分别增长10.1%和29.8%。“互联网+龙门”树立国内智慧旅游新标杆。规模以上文化企业增长1.25倍。我市成为全省唯一的国家体育产业联系点城市。信息服务业营业收入、利润分别增长27%和22.8%。特色高效农业态势良好。农业增加值增速居全省第一。签约农业项目249个、总投资381.4亿元。华大基因河南（洛阳）农业创新中心挂牌成立，首批牡丹基因组研究成果获得5项世界第一。新增市级农业产业化集群12个。产业集聚能力不断提升。全市产业集聚区开工亿元以上主导产业项目163个；完成固定资产投资1691.6亿元，增长18.3%。我市入选国家电子功能材料产业区域集聚发展试点。

（三）完善功能打基础、强支撑，城乡一体化水平不断提升。中心城区功能不断完善。应天门环线等工程建成投用，城市环线等项目加快实施，瀛洲东路等项目开工建设。新建、提标污水处理厂26个，铺设污水管网126公里。新增供热面积780万平方米。建成电网工程700余项。引黄入洛工程全线贯通。洛河东湖、伊河东湖主体工程完工。我市顺利通过国家卫生城市复审验收。数字化城市管理系统建设项目荣获中国人居环境范例奖。棚户区改造稳步实施。全年开工1290万平方米，竣工730万平方米，2.2万户群众喜迁新居。综合交通体系建设步伐加快。尧栾西高速、洛济高速等项目前期工作取得实质性进展，连霍高速洛阳境改扩建等工程稳步推进，洛吉快速通道二期建成通车。洛阳至曼谷国际航班顺利开通，航空口岸实现常态化运营，全年机场旅客吞吐量74.3万人次。农村生产生活条件不断改善。前坪水库开工建设。美丽乡村建设扎实推进，硬化通组通户道路2011公里，改造农村危房2.62万户，解决了32.4万人口的饮水安全问题。生态环境持续改善。截流河渠排污口59个，拆除燃煤锅炉43台，淘汰黄标车2.3万辆，完成310万千瓦燃煤机组超低排放治理。造林绿化50万亩、抚育改造森林87.2万亩。

（四）凝心聚力抓改革、扩开放，发展动力活力持续增强。重点领域改革扎实推进。53个市直部门权责清单编制完成。全面推行“三证合一、一照一码”改革，新增市场主体7.1万户。支持有色矿业集团、中硅高科等开展混合所有制改革试点工作。驻洛央企“三供一业”分离移交工作顺利实施。开放招商成果丰硕。签订重大招商引资合同405个，总投资2463.8亿元，79个10亿元以上重大项目、11个境内外500强企业落户洛阳。瞪羚谷加速器等洛深合作项目启动实施。一拖东方红公用型保税仓库正式挂牌，洛阳至青岛港口货运专列成功首发。创新创业成效显著。新建企业研发平台168家，培育国家级众创空间2家、占全省的三分之一。空空导弹研究院制造团队代表我国取得世界技能大赛金牌零的突破。洛阳高端装备制造产业基地入选国家高新技术产业化基地。高新技术产业增加值增长14.4%，高于全市工业4.7个百分点。大学科技园入选国家级技术转移示范机构、全国青年创业示范园区。我市荣膺全国小额担保贷款重点调度城市、全省创业型试点城市。中信重工“双创”模式受到李克强总理的充分肯定。

（五）千方百计办实事、惠民生，人民生活水平不断提高。省定市定实事圆满完成。新增城镇就业12.98万人、再就业3.8万人。建成各类保障房1.18万套。完成整村推进125个村、搬迁贫困群众3.1万名，5.95万名群众实现脱贫。实施中小学改扩建项目614个，新建改扩建幼儿园186所，在全省率先实现义务教育“四免一补”。提高城镇居民医疗保险财政补助标准和新农合人均筹资额，大病保险制度实现城镇居民全覆盖。新建农村老年幸福院228个。第五人民医院新区医院、正骨医院康复院区建成投用。启动城市公立医院药品零差率销售和分级诊疗试点。“互联网+医疗”平台上线运行。承办了国足热身赛等一批高水平赛事。110联动服务平台办结率、满意率均达95%以上。在全省率先推行安全生产信用体系建设，加强和改进信访稳定工作，深入推进“平安洛阳”建设，社会形势和谐平稳。

（六）从严从实转作风、提效能，政府自身建设进一步加强。“三严三实”专题教育扎实开展。依法行政全面推进，法治政府建设取得新成效。自觉接受人大及其常委会监督，积极支持政协参政议政，办理人大代表建议347件、政协提案428件，满意率99%以上。持续推进反腐倡廉建设，一批违法违纪典型案件受到严厉查处。军民融合发展质量不断提升，国防动员和后备力量建设取得新进步，军政军民团结进一步加强，顺利通过全国双拥模范城七连冠考核。同时，民族宗教、外事侨务、对台、统计、审计、人防、气象、地震、档案、史志、残疾人工作、妇女儿童事业等都取得了新的成绩。

五年砥砺奋进、五年开拓创新、五年辉煌成就，为洛阳“十三五”发展奠定了坚实基础。这些成绩的取得，是党中央国务院、省委省政府和市委正确领导的结果，是市人大、市政协监督支持的结果，更是700万洛阳人民团结拼搏的结果。在此，我代表市人民政府，向辛勤工作在各条战线上的全市人民，向人大代表、政协委员，向各民主党派、人民团体，向驻洛部队、武警官兵和公安干警，向离退休老领导、老同志，向所有关心支持洛阳发展的社会各界人士，表示衷心的感谢并致以崇高的敬意！

回顾五年来的工作，我们深深地体会到：必须将发展这个第一要务作为一切工作的基础和关键，做大做强实体经济，提升中原经济区副中心城市的带动力和辐射力；必须将创新驱动作为洛阳经济发展的第一动力源，把雄厚的科技资源转化为强大的发展优势；必须将改革开放作为推动洛阳发展的根本动力，靠改革拓空间、添活力，靠开放扩增量、强后劲；必须将统筹城乡发展作为全面建成小康社会的重要举措，加快新型城镇化和新农村建设；必须将改善民生作为政府工作的出发点和落脚点，使全市人民共享改革发展成果；必须将创先争优作为政府工作的基本目标，力争各项工作在全国、全省有位次、有影响。这些宝贵经验是洛阳人民在艰苦实践中获得的智慧结晶，我们要倍加珍惜、大力弘扬！

与此同时，我们也清醒地看到，当前洛阳正处于爬坡过坎、转型跨越、蓄势崛起的关键时期，前进的道路上还面临不少困难和挑战。一是产业结构不优，体制机制不活，产学研融合程度不高，产业转型、深化改革的任务十分繁重；二是城乡二元结构矛盾突出，脱贫攻坚任务艰巨；三是教育、医疗、就业、住房等关系群众切身利益的问题尚未得到妥善解决；四是环境问题较为突出，生态建设任重道远；五是政府工作人员的履职能力、勤政廉政意识和依法行政水平有待进一步提升。对于以上问题，我们决不回避、决不推卸，将尽最大努力认真解决。

二、“十三五”主要目标任务

“十三五”时期是全面建成小康社会的决胜阶段，也是洛阳建设中原经济区副中心城市最关键的时期。站在“十二五”铸就的辉煌起点上，我们必须准确把握经济发展新常态带来的各种变化和洛阳在全省、全国的地位，以创新、协调、绿色、开放、共享五大发展理念为引领，以构建现代创新体系、现代产业体系、现代市场体系、现代城镇体系、现代基础设施体系、文化传承创新体系、生态环境建设体系、开放体系、公共服务体系为支撑，精心绘就“十三五”宏伟蓝图，全力开创洛阳发展新局面。

“十三五”全市经济社会发展的主要预期目标是：确保经济发展、人民生活、城镇化水平和生态文明程度高于全省平均水平，科技创新能力更强，巩固提升中原经济区副中心城市地位，在全省率先全面建成小康社会。

——综合实力大幅提升。经济总量年均增长9%。到2020年，全市地区生产总值和城乡居民人均收入比2010年翻一番以上，经济总量稳居中原经济区第2位、占全省的比重达到10%以上。

——发展活力明显增强。全社会研发投入占GDP的比例达到2.5%，建成国家自主创新示范区；全方位、宽领域、高水平的开放格局基本形成；重点领域和关键环节改革取得决定性成果，形成系统完备、科学规范、运行有效的制度体系。

——城乡发展更加协调。完成国家新型城镇化综合试点建设任务，构建现代城镇体系，形成中心城市、县城组团、特色小镇、美丽乡村“四级联动”的城乡一体发展新格局。到2020年，全市常住人口城镇化率达62%。

——文化软实力全面提升。文化产业快速发展，华夏历史文明传承创新特色突出，公共文化服务体系较为完善，文明城市建设水平保持全国先进位次，成为影响广泛的中原经济区文化中心和国际文化旅游名城。

——生态环境显著改善。到2020年，森林覆盖率达到50.5%以上；城市区人均水面面积达到8平方米；城市环境空气质量平均达标天数比例明显提高，城镇集中式饮用水水源稳定达标；绿色低碳发展取得新成效，成为中西部地区低碳生态城市建设样板。

——民生质量普遍提高。就业岗位持续稳定增加，覆盖城乡的社会保障体系进一步完善，教育、医疗、文化、养老等基本公共服务均等化程度提高，城乡居民人均收入增幅高于生产总值增幅，农村贫困人口全部脱贫，全面建成小康社会。

新的目标催人奋进，我们要自我加压、负重拼搏，全力保持稳定向好的发展态势。“十二五”以来，我市经济逆势回升的态势持续显现，特别是在去年全国、全省经济增速普遍放缓的大背景下，我市经济增速逐季攀升，重回全省第一方阵，已经具备了在新起点上晋位争先的基础。只要我们团结一心、务实重干，就一定能够在全省第一方阵站稳脚跟、再攀高峰。

新的征程扬帆启航，我们要乘势而上、主动作为，牢牢把握十分难得的历史机遇。国家促进中部地区全面崛起、推进“一带一路”建设，推动大众创业万众创新，全面实施“互联网+”行动计划、“中国制造2025”等战略，为我市加快产业转型升级、抢占发展制高点带来了重大机遇。只要我们敢于抢占先机、厚植优势，就一定能够在新一轮竞争中实现跨越赶超！

新的蓝图已经绘就，我们要矢志不渝、久久为功，努力实现全面小康的美好愿景。“十三五”规划描绘了未来五年全面建成小康社会的美好蓝图，是指引洛阳今后五年发展的行动纲领，承载着700万人民对幸福生活的梦想与期待。只要我们紧紧团结和依靠全市人民，凝聚干事创业的巨大能量，就一定能够在全省率先全面建成小康社会，一个经济更加繁荣、社会更加和谐、生态更加优美、人民更加幸福的新洛阳必将崛起在中原大地！

三、2016年重点工作

2016年是全面实施“十三五”规划、决胜全面小康的开局之年。今年市政府工作的总体思路是：全面贯彻党的十八大和十八届三中、四中、五中全会精神，认真落实党中央国务院、省委省政府和市委决策部署，自觉践行创新、协调、绿色、开放、共享五大发展理念，更加注重稳增长、促改革、调结构、强基础、惠民生、防风险综合平衡，加快构建现代创新体系、现代产业体系、现代市场体系、现代城镇体系、现代基础设施体系、文化传承创新体系、生态环境建设体系、开放体系、公共服务体系九大体系，确保“十三五”良好开局，为实现“四高一强一率先”的奋斗目标奠定坚实基础。

今年全市经济社会发展的主要预期目标是：地区生产总值增长9%；一般公共预算收入增长8%；固定资产投资增长16%；社会消费品零售总额增长12%；外贸进出口总额增长7%；居民消费价格指数控制在3%左右；节能减排完成省下达任务；城乡居民人均收入增速高于GDP增速。

实现上述目标，必须准确把握好以下几个方面：一要着力实施创新驱动发展战略，始终抓紧牵动经济社会发展全局的“牛鼻子”。以创建国家自主创新示范区为契机，以科技创新为引领，全面推动产业创新、企业创新、市场创新、产品创新、业态创新、管理创新，培育壮大发展新动能。二要着力增强发展的整体性协调性，积极推动城乡居民同步全面小康。立足中原经济区发展的大格局，加强交通、生态、产业等方面的对接融入，加快构建现代城镇体系，坚决打赢脱贫攻坚战。三要着力推进人与自然和谐共生，加快构建城乡一体的良好生态体系。牢固树立尊重自然、顺应自然、保护自然的理念，推动形成绿色发展方式和生活方式，让河洛大地天更蓝、山更绿、水更清、环境更美好。四要着力形成对外开放新体制，不断提升对外开放的质量和水平。深度融入“一带一路”战略，积极承接国内外产业转移，不断扩大洛阳作为丝绸之路经济带主要节点城市的开放优势。五要着力践行以人民为中心的发展思想，让改革发展成果更多更公平地惠及广大群众。始终把人民对美好生活的向往作为政府工作的奋斗目标，大力推进以改善民生为重点的社会建设，把群众最关心、最直接、最现实的利益问题解决好。

重点要抓好以下八个方面的工作：

（一）强力推进供给侧结构性改革，厚植产业竞争新优势。加快构建现代产业体系，努力提高供给体系的质量和效率，为稳增长、调结构提供强劲动力。全年计划实施重大项目1248个，完成投资2500亿元以上。

推动实体经济健康发展。一是稳企业。完善领导分包、联审联批等长效机制，支持形势好的企业扩大产能，多做贡献；帮助困难企业解困突围，防范化解债务风险，做好稳岗就业工作。鼓励传统行业开发新产品、新工艺，挖掘更多盈利点。二是降成本。全面实施降低实体经济企业成本行动，打好降成本、减税费“组合拳”，为实体经济送去含金量更高的“政策红包”。三是解难题。深化产销、银企、用工等对接，抓好政府增信基金、应收账款质押贷款等工作，创新股权和知识产权质押贷款等金融服务。确保全年银企洽谈会签约资金落实率超过85%，企业还贷周转金年周转量40亿元以上。四是去库存。完善政策措施，鼓励农民和外来人员进城购房，防控商业地产风险。

提升工业发展质量效益。实施工业强基工程，推动工业向产业链和价值链高端延伸。全年计划实施重大工业结构调整项目358个，完成投资529亿元。一是改造提升传统产业。围绕工业化信息化融合，加快中信重工、一拖集团国家互联网与工业融合创新试点建设，启动实施十大智能制造示范工程，创建一批“两化”融合示范企业。围绕石化煤化融合，开工建设洛阳石化1800万吨炼油扩能改造及配套项目等，进一步拉长产业链条，努力打造中西部地区最大的石化煤化基地。围绕制造业服务业融合，抓好LYC轴承重大工程机械配套项目产业化等重点项目，支持重点企业由设备制造商向系统服务商转变。围绕军民融合发展，加快项目配套和产业链延伸，争创国家军民融合创新示范区。围绕传统制造业绿色改造，大力推广高效电机、节煤和资源综合利用设备产品，确保中信重工节能环保装备产业化基地等项目竣工投产。二是培育壮大战略新兴产业。围绕机器人及高端装备制造业，全面实施“131”重点企业培育工程，着力突破机器人共性核心技术，突出重点产品应用示范和产业化，加快建设全省机器人及智能装备产业基地。围绕电子信息产业，抓好河南移动（洛阳）大数据云计算中心、洛阳微电子产业园等重点项目，力争心里程集团平板电脑等项目竣工投产。围绕新材料产业，加快国家电子功能材料产业区域集聚发展试点建设，重点实施中航锂电产业园三期、正威有色科技城等项目。围绕新能源及新能源汽车产业，支持骨干企业突破核心技术，扩大优势产能，加快实施阿特斯太阳能光伏3吉瓦全产业链大型制造基地、东风新能源12万台新能源汽车等重点项目。三是提升产业集聚区发展水平。扎实推进管理体制套合改革，加快培育百千亿级优势产业集群。全年计划实施亿元以上主导产业项目470个，固定资产投资增长18%以上，规模以上工业主营业务收入增长12%以上，力争新增一星、二星级产业集聚区各3家，进入全省“十强、十快”产业集聚区3家。

促进服务业提质增效。坚持生产性服务业和生活性服务业融合发展，促进现代服务业提档升级。全年计划实施现代服务业项目200个，完成投资400亿元。一是做大做强文化旅游业。围绕构建文化传承创新体系，推动文化旅游深度融合发展，谋划实施文化旅游产业示范工程和牡丹文化产业提升工程。大力发展“互联网+旅游”，加快智慧旅游试点建设，完善旅游公共服务体系。抓好白云山、万安山整体开发等一批重大项目，做好栾川、嵩县国家全域旅游示范区创建工作，培育特色文化旅游产品，科学规划推介精品旅游线路，积极申报中国国际历史文化特色旅游目的地，力争全年接待游客1.14亿人次、旅游总收入860亿元。加快建设国家级文化科技融合示范基地，积极创建国家级文化产业示范基地，提升特色文化产品竞争力。办好第34届中国洛阳牡丹文化节，积极引进品牌展会，培育地方特色展会。二是改造提升商贸物流业。大力发展楼宇经济，稳步推进大型综合商业设施建设，16个“两区”计划完成投资130亿元。积极推进电子商务与物流快递协同发展试点城市建设，抓好中储综合物流园等重点物流项目。三是培育壮大信息服务业。积极引进知名电商企业，大力培育特色电商平台，加快农超网等电商产业园建设，力争电子商务交易额增长25%以上。大力发展软件及服务外包、大数据、云计算、物联网等新兴业态，加快实施中科信息产业研究院等项目。四是积极发展健康养老和体育产业。抓好国家养老服务业综合改革试点工作，稳步推进国家级居家式新型养老示范基地建设，大力发展医养结合、社区养老、居家养老。推进国家体育产业联系点城市建设，谋划建设一批体育产业基地。

（二）培育壮大现代农业，开创“三农”工作新局面。加大强农惠农富农力度，积极推动农业现代化。大力发展现代特色高效农业。抓好高标准粮田和粮食流通能力建设，粮食总产稳定在40亿斤以上。新发展牡丹3万亩，稳定蔬菜、水果种植面积和水产养殖面积，大力发展特色农产品加工业，推动特色杂粮、中药材、花卉苗木、畜牧养殖等优势产业向规模化、标准化、品牌化发展。加快实施都市生态农业提升工程，重点培育100家以上休闲农业精品园区。发展壮大林业经济。做大做强10个农业产业化集群，力争创建省级以上出口农产品质量安全示范区4个以上。提升农业支撑保障能力。加快推进前坪水库、小浪底南岸灌区等工程，新增有效灌溉面积3万亩，发展节水灌溉3万亩，治理水土流失150平方公里。完成9座小型水库除险加固。新增农机动力5万千瓦以上。加大农业科技推广服务力度，探索建立农产品质量安全追溯体系。培育新型农业经营主体。实施“百社百场育强工程”，新发展市级农业产业化龙头企业20家、产业化集群5个。大力培育新型职业农民。积极推进土地托管新模式，新增农村土地托管面积40万亩。强力推进脱贫攻坚。认真落实“六个精准”，抓好贫困村道路畅通等六项重点工程，实施整村推进130个村，完成扶贫搬迁5000户，确保6.8万人脱贫、214个贫困村摘帽。

（三）加快实施创新驱动发展战略，培育经济发展新动力。坚持政府主导、企业主体、项目带动、人才支撑，积极构建现代创新体系，全面推进大众创业万众创新，着力打造中原经济区产业创新中心。加强载体平台建设。扎实推进郑洛新国家自主创新示范区建设。积极申报国家“两创”示范城市。支持50家重点企业设立研发平台，围绕重点产业建设一批公共技术服务平台。加快洛深创新生态城建设。积极推进中科院等在洛研发机构建设发展，开工建设清华启迪科技新城等项目，争取引进更多的新型研发机构和产业基地。全年培育众创空间3个，新建各类研发机构18个、市级以上科技企业孵化器2个、高新技术企业和创新型试点企业15家。深化产学研用融合。探索建立政府、企业、高校和科研院所协同创新推进机制，加快建设众创孵化空间和创新创业投融资平台。支持有条件的重点企业牵头组建产业技术创新战略联盟。启动科技大市场建设，建成网上技术交易平台，打通成果转化的“最后一公里”。实施国家知识产权战略行动计划，探索开展专利质押融资、专利保险。深化科技体制改革，落实技术交易减免税等优惠政策，用好科技型中小企业贷款风险补偿资金池，探索设立天使投资基金，激发科研人员创新创业潜力。加快汇聚高端人才。大力实施“河洛英才计划”，打造洛阳人才创客空间，推动高端人才创新创业，全年培育省级引智示范基地1－2家、市级1－2家。推动创业带动就业。支持洛阳大学科技园等创建国家级创业孵化示范基地，争创3家省级创业孵化示范基地，实现县级创业孵化园县域全覆盖。实施创业贷款资金扶持计划，实行“创贷+商贷”新模式，发放创业担保贷款15亿元。支持高校众创空间建设。新增城镇就业10万人、再就业3万人。

（四）科学推进新型城镇化，构建城乡统筹新格局。做好规划、建设、管理三篇文章，以人为核心推进新型城镇化，着力构建现代城镇体系和现代基础设施体系。提高城市规划水平。做好第五期城市总体规划修编工作，提升区域性规划和专项规划编制水平。全面开展城市设计，统筹城市建筑布局，体现城市传统文脉、建筑肌理和时代风貌。加快市政工程建设。突出抓好古城路快速化，王城大道与古城路、隋唐城路互通立交，珠江路下穿洛河隧道等十大城建重点工程；城区主干道及重要节点绿化、路灯亮化节能改造等十大城市功能提升工程。加快新街跨洛河大桥、滨河南路等项目建设，确保炎黄路、开拓大道跨伊河大桥、引洛济伊等一批项目牡丹文化节前建成，伊滨区第二污水处理厂上半年竣工，城市生活垃圾综合处理园区9月底前点火试运行，力争环城路全线贯通，确保市规划馆建成开放。改善群众居住条件。加快实施96个棚户区改造项目，开工安置房2万套、竣工3万套，安置群众2.6万户。建立购租并举住房制度，提高棚改货币化安置率。全年计划完成房地产投资280亿元，新开工1000万平方米，竣工600万平方米。彰显历史文化特色。加快实施偃师商城、汉魏洛阳城遗址、九洲池和应天门保护展示等工程，开工建设二里头夏都遗址博物馆，确保老城历史文化街区一期建成投用。推进仓窖博物馆、丝绸之路博物馆和民办博物馆建设。强化基础保障能力。加快环城燃气管网建设，抓好洛阳至嵩县、栾川天然气管线前期工作。加快实施吉利区2×350兆瓦热电联产燃气电厂项目，争取万基控股2×600兆瓦机组、220千伏宜阳南等18项输变电工程开工建设，大鱼沟抽水蓄能电站项目完成核准。新增供热面积350万平方米。抓好地下综合管廊、停车场等建设。完善综合交通体系。加快三门峡至洋口港铁路、城市轨道交通（地铁）、龙门高铁枢纽、动车运用所前期工作，争取郑登洛城际铁路、焦济洛城际铁路、尧栾西高速、洛济高速洛阳段、龙门长途客运站等项目尽快开工，加快建设310国道洛阳段改建、洛吉快速通道黄河大桥等项目，确保二广高速洛阳瀍河互通立交项目牡丹文化节前建成通车。新建改建县乡道路200公里。积极推进机场改革，新开通国内外航线3－5条，旅客吞吐量突破100万人次。提高城镇管理水平。加快数字化城市管理系统二期工程建设，实现市、县数字化城市管理全覆盖。抓好智慧城市云平台建设，提升交通智能化管理水平。积极推进城市执法体制改革。实施提升县级城市管理水平三年行动计划和县城综合改造提升行动。加快重点镇、景观旅游名镇和中心镇（村）建设，突出抓好十大特色镇建设工程。加快建设美丽乡村。建成66个市级美丽乡村试点，确保765个以上村达到提升村创建标准、510个以上村庄完成示范村建设目标。加强传统村落保护。改造农村危房5000户。硬化通组通户道路2000公里。加快农村电网升级改造。

（五）坚定不移推进绿色发展，建设生态美丽新洛阳。以更大的力度保护和改善生态环境，打造具有洛阳特色的生态环境建设体系。狠抓重点污染治理。彻底拆除集中供热区域燃煤锅炉，实现全市火电行业达标排放，加强工业废气治理、黄标车淘汰、油气回收治理工作，全面控制扬尘污染。购置清洁能源公交车辆310辆。做好重污染天气应急工作，有效遏制雾霾天气频发，力争空气质量明显改善。全面实施城市河流清洁行动，继续开展城市河渠整治及排污口截流，治理河道30公里。确保城镇污水处理设施全面达到一级A排放标准，所有产业集聚区实现污水管网全覆盖。保障城市饮用水水源安全。健全生态环境损害责任追究机制，严厉打击环境违法行为。提升生态绿化水平。加快实施隋唐城国家考古遗址公园（万亩园林）、园林绿化生态提升等项目，提升南部山区旅游通道绿化水平，着力打造城市绿色屏障。规划建设一批城市街头小游园，打造一批特色鲜明的城市景观大道，持续增加城市绿量。实施林业生态建设提升工程，抓好宜林荒山绿化和林业生态圈建设，完成造林绿化35万亩以上、森林抚育改造160万亩。推进生态水系建设。积极创建全国水生态文明试点城市，开工建设“三个千万方”调蓄工程和“三个百里”生态廊道，加快推进伊洛河水生态文明示范区等重点水系工程，打造水文化生态文明核心区。全面完成华夏文明第一河示范段、洛河东湖、伊河东湖、伊洛运河等建设任务，确保故县水库引水工程9月底前建成投用。加强湿地保护。推动低碳循环发展。加快建设中欧低碳生态城市合作项目综合试点城市、国家循环经济示范城市、“城市矿产”示范基地和餐厨垃圾综合利用试点城市，积极推进海绵城市建设和绿色建筑产业化。抓好重点节能项目，开展节能环保产品推广示范。积极推进碳交易市场建设。落实最严格的耕地保护和水资源管理制度，提高能源资源综合利用效率。积极开展生态创建活动。

（六）持续深化重点改革，激发体制机制新活力。全面落实国家、省改革部署，着力破解瓶颈障碍，构建现代市场体系，增强发展内生动能。加快新型城镇化综合试点建设。完善中心城区、县城落户措施，维护进城落户农民土地承包权、宅基地使用权、集体收益分配权，有序推进农业转移人口市民化，力争全年进城落户3万人以上。做好国家新型城镇化融资试点工作，全面实施综合试点融资规划，用好900亿元城镇化发展基金。大力推广PPP模式。扎实推进水电气热等重点领域价格改革。深化国企国资改革。完善国有资产监督管理体制，组建改建国有资本投资运营公司，支持市属国有企业发展混合所有制经济，探索实行混合所有制企业员工持股，深入推进企业内部三项制度改革试点、驻洛央企“三供一业”分离移交工作。推动金融改革创新。大力引进中原银行等金融机构，加快洛阳银行上市进程，确保农信社全部组建农商行。支持保险机构开展贷款保证保险和贸易信用保险，争取利用保险产品融资8亿元以上。探索设立“新三板”专项基金，推广融资租赁、集合票据、中小企业私募债等多种融资工具，探索股权众筹、互联网金融等中小企业直接融资新模式。新增融资500亿元，实现12家公司上市（挂牌）或再融资。深化农村改革。基本完成农村土地承包经营权确权登记颁证工作。积极实施“增力强基”工程，稳步推进农村集体经济组织股份合作制改革。鼓励支持嵩县、栾川等试点探索农村产权制度改革。扎实推进栾川全国集体林业综合改革试验示范区建设。发展壮大民营经济。实施“小巨人”企业培育工程，新上千万元以上民营经济项目440个。持续深化商事制度改革，巩固扩大“三证合一”改革成果，确保实现“一照一码”，新增各类市场主体5万户。

（七）加快推进全方位对外开放，打造内陆开放新高地。坚持引进来和走出去相结合，加快建设开放体系，提升外向型经济发展水平。构建双向开放平台。积极融入“一带一路”国家战略，抓好一拖集团中白工业园研发中心、中信重工柬埔寨水泥厂等重点项目，鼓励中航锂电、中油一建等优势企业扩大国际产能合作。借力郑欧国际铁路货运班列，加快铁路口岸建设，积极申建海关特殊监管区域，争取西工综合保税区年内获批开建，提升航空、铁路、公路等口岸功能。积极申建自贸区洛阳片区、国家产城融合示范区。围绕创建国家服务外包示范城市，争创3－5家省级服务外包示范园区，建成国家服务外包交易中心洛阳平台，推动符合条件的产业集聚区争创省级开发区。加强与国内外友好城市的经贸、科技、人才等交流合作。深化重点产业招商。借助河南投资贸易洽谈会、拜祖大典、牡丹文化节等开放招商平台，扎实推进专题专项招商，持续开展十大重点产业招商行动，提高合同履约率和项目开工率，力争引进60个10亿元以上重大产业项目。推动外贸持续增长。调整出口商品结构，培育一批出口型龙头企业，引进一批出口导向型项目，支持企业拓展国际新兴市场。鼓励对外工程承包与劳务合作，支持出口基地服务平台和国家级对外劳务合作服务平台建设。

（八）切实保障和改善民生，实现人民福祉新提升。加大民生投入，办好民生实事，完善公共服务体系，不断提高群众的获得感！让孩子们享有更优质的教育。落实中小学布局专项规划，加快实施第二期学前教育三年行动计划，新建、改扩建中小学249所、幼儿园148所。推进“全面改薄”和城镇义务教育资源扩充计划，全面消除市区学校“大班额”和县镇学校“超大班额”。积极稳妥推进农村教学点布局调整工作，实施农村全科教师培养计划，改善贫困地区办学条件。加快推进普及高中阶段教育。实施职教攻坚二期工程，建成1－2家示范性职业教育集团。力争洛阳职业技术学院开工建设、河科大二期年底建成投用。让群众享有更便捷的医疗卫生服务。深化城市公立医院综合改革，在公立医院全面施行药品零差率销售，全面开展高血压、糖尿病分级诊疗试点工作。抓好河科大一附院病房楼等重点项目，新增床位1300张，力争第一中医院新区医院开工建设。完善基层医疗卫生服务体系，全面实施国家基本公共卫生和重大公共卫生服务项目。推广应用全民健康信息保障工程。坚持计划生育基本国策，落实全面两孩政策。让群众享有更丰富的文化生活。完善公共文化服务体系，积极实施各类文化惠民工程。加快市文化馆、少儿图书馆、非遗展示馆和10个基层综合性文化服务中心建设，确保市曲剧团、豫剧团多功能演艺厅尽快建成投用，国家公共文化服务体系示范区、省全民健身示范市顺利通过验收。完善公共体育健身设施网络，广泛开展全民健身活动。让群众享有更完善的社会保障。推进城镇居民基本医疗保险和新型农村合作医疗整合，统一城乡居民基本医疗保险制度。稳妥推进机关事业单位养老保险制度改革。认真落实困难残疾人生活补贴、重度残疾人护理补贴和计划生育特殊家庭养老扶助制度，适时提高城乡困难群众救助标准。完善“五险合一”模式，抓好城镇居民大病保险试点工作，提高异地就医结算城市辐射能力。适度提高新农合支付比例和县级医院住院补偿比例，降低大病保险起付线。新建农村幸福院200个，建成智慧社区养老服务中心试点10个，全市社区养老服务中心覆盖率达到80%以上。让群众享有更和谐的社会环境。严格落实安全生产责任制，持续开展重点行业、重点领域隐患排查治理，在全市所有街道和社区建成微型消防站，坚决遏制重特大安全事故发生。健全食品药品安全监管体系，落实最严格的全程监管制度。深化平安洛阳建设，构建立体化社会治安防控体系，严厉打击各类违法犯罪活动。依法化解社会矛盾，全力维护群众合法权益，坚决守住不发生系统性、区域性风险的底线。

高度重视国防教育，增强全民国防意识。积极支持深化国防和军队改革，做好新形势下的国防动员、民兵预备役和双拥共建工作，深入推动军民融合发展。支持工会、共青团、妇联等工作。做好民族宗教、外事侨务、对台、统计、审计、人防、气象、地震、档案、史志、残疾人事业等工作。

四、建设人民满意政府

以新的发展理念引领政府自身建设，加快推动政府治理体系和治理能力现代化，树立为民务实清廉的政府形象。

强化法治思维，全力打造法治政府。全面落实法治政府建设实施纲要，大力推进依法治市，引导全社会尊法学法守法用法，按规律、按程序办事，提高依法行政水平。加强重点领域立法，健全依法决策机制，完善重大行政决策程序，推动政府法律顾问制度全覆盖。全面落实行政执法责任制，推进服务型行政执法建设，提升行政复议、仲裁化解社会矛盾纠纷的法治化水平。严格执行人大及其常委会决定，提高人大代表建议和政协提案办理质量，自觉接受人大及其常委会法律、工作监督和政协民主监督，加强与民主党派、工商联、人民团体的沟通协商，重视社会监督和舆论监督。深入推进政务公开，让权力运行更加公平公正、阳光透明！

强化服务意识，加快转变政府职能。深入推进放、管、服改革，大刀阔斧简政放权，清理规范中介服务事项，为创业创新铺路搭台、为企业发展松绑减负。完善政府守信践诺机制，加强全社会诚信建设。巩固和完善权责清单制度建设，确保3月底前县（市、区）级政府编制公布“两单”。推进智慧政务建设，大力开展“互联网+益民服务”行动，抓好行政审批标准化试点，加快推行联合审批、网上审批，构建市、县、乡三级审批便民服务系统，让信息多跑路、群众少跑腿、企业不费事。大力优化发展环境，鼓励激励全民创业，营造重商、扶商的浓厚氛围。

强化担当精神，锤炼勤勉务实作风。严格执行中央八项规定精神，自觉践行“三严三实”，深入开展“两学一做”学习教育，杜绝“四风”反弹回潮。扎实开展懒政怠政为官不为问责年活动，做到有令则行、行则迅速，有禁则止、止则彻底。完善公务员培训教育制度，提高公务员队伍整体素质和履职能力。积极推行“一线工作法”，把更多的目光投向基层，把更多的力量沉向基层。完善政务督查机制，建立目标任务、完成时限、责任人员“三个清单”，推动政府每件工作都善始善终、每项政策都落地生根。

强化纪律观念，狠抓反腐倡廉建设。坚持把纪律和规矩挺在前面，严格执行廉洁自律准则、党纪处分条例等各项规定，全面落实党风廉政建设主体责任和“一岗双责”。健全惩治和预防腐败体系，推动监察和审计全覆盖，加大对重点领域和关键环节的监督力度，从源头上遏制和预防腐败行为。注重家教、注重家风，以良好的家风带动政风。严厉查处群众身边的不正之风和腐败问题，以坚定的意志和决心反腐惩恶，以反腐惩恶的成效保障发展、取信于民，打造风清气正的政务环境。

各位代表！雄关漫道真如铁，而今迈步从头越。让我们紧密团结在以习近平同志为总书记的党中央周围，在省委、省政府和市委的坚强领导下，团结拼搏，务实奉献，为早日实现“四高一强一率先”的宏伟目标而努力奋斗！
